# Dogs with leishmaniosis: how are we managing proteinuria in daily practice? A Portuguese questionnaire-based study

**DOI:** 10.1186/s13071-022-05222-w

**Published:** 2022-04-11

**Authors:** Marta Monteiro, Sara Prata, Luís Cardoso, Isabel Pereira da Fonseca, Rodolfo Oliveira Leal

**Affiliations:** 1 Hospital Escolar Veterinário, Faculdade de Medicina Veterinária, Universidade de Lisboa, Lisbon, Portugal; 2 Department of Veterinary Sciences, and Animal and Veterinary Research Centre (CECAV), University of Trás-os-Montes and Alto Douro (UTAD), Vila Real, Portugal; 3 Centro de Investigação Interdisciplinar em Sanidade Animal (CIISA), Faculdade de Medicina Veterinária, Universidade de Lisboa, Lisbon, Portugal; 4 Laboratório Associado Para Ciência Animal e Veterinária (AL4AnimalS), Lisbon, Portugal

**Keywords:** Antiproteinuric treatment, Canine leishmaniosis, Dog, Immunosuppressants, Questionnaire-based study

## Abstract

**Background:**

Proteinuria is a common finding in dogs with leishmaniosis. Although antileishmanial therapeutic protocols are widely implemented, little information is available on which treatments are most adequate for identifying proteinuria in patients with canine leishmaniosis (CanL), especially regarding the use of immunosuppressants. The aim of this study was to explore the current paradigm regarding the antiproteinuric approach adopted by veterinary practitioners in Portugal to treat dogs with CanL.

**Methods:**

A questionnaire-based study was developed using Google Forms®. The questionnaire presented a number of different hypothetical scenarios of CanL, and the topics surveyed included the general features of the respondents and the protocols preferred by these respondents to manage proteinuria in the presented scenarios, including choice of therapeutic drugs, appropriate diet and use of immunosuppressants, in dogs with immune-mediated glomerulonephritis. The questionnaire was internally prevalidated and diffused online over a 2-month period through Portuguese veterinary social networking groups, and data were collected for descriptive analysis.

**Results:**

A total of 86 veterinary practitioners responded to the survey. When exposed to theoretical scenarios of proteinuria in dogs with CanL at stages IIb, III and IV (LeishVet guidelines), 16.3%, 62.8% and 93.8% of the respondents, respectively, answered that they would treat it. The dog was started on a renal diet as therapy by 28.6%, 83.3% and 97.4% of respondents, respectively. Angiotensin-converting enzyme inhibitors (ACEI) were prescribed by 100%, 85.2% and 78.9% of respondents as first-choice drugs for CanL at stages IIb, III and IV, respectively, with ACEI used in monotherapy by 64.3%, 40.7% and 46.1%. In comparison, protocols using ACEI in combination with other compounds were chosen by 7.1%, 33.3% and 39.5% of respondents, and combination therapy which did not include ACEI was the choice of 0.0%, 12.9% and 14.5%. Regarding immunosuppressants, 44.2% of the respondents answered they would prescribe them, with 97.4% electing for prednisolone and 5.3% choosing mycophenolate mofetil.

**Conclusions:**

Among the veterinary practitioners who responded, proteinuria treatment was considered since stage IIb CanL, although implementation of a therapeutic approach was more evident in advanced CanL stages. ACEI were the first-choice drugs, particularly for the treatment of stage IIb CanL; in advanced stages, a combination of antiproteinuric drugs was more often used. Immunosuppressant use was controversial, although when applied, prednisolone was the preferred choice. These findings reinforce the small body of evidence that supports the use of such drugs and the need to further explore their role in CanL.

**Graphical Abstract:**

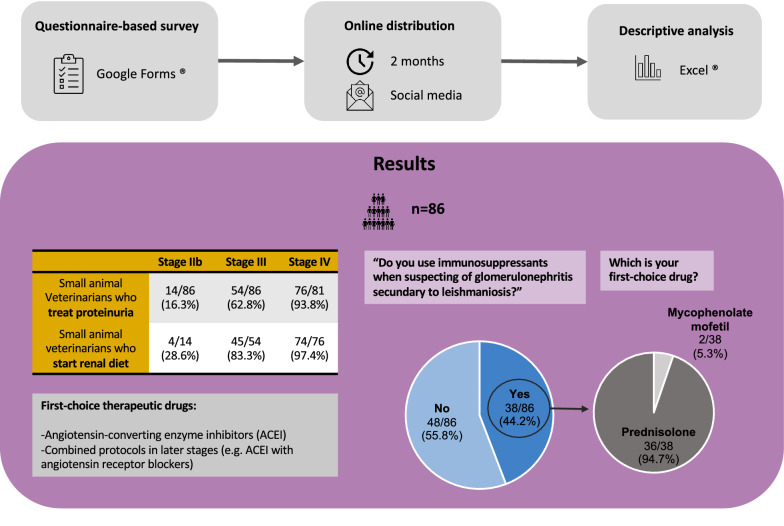

**Supplementary Information:**

The online version contains supplementary material available at 10.1186/s13071-022-05222-w.

## Background

Canine leishmaniosis (CanL) is an endemic disease in many countries across the world [1–3]. Renal compromise is one of the most common and important complications in dogs with leishmaniosis [4–6] and is often detected through the presence of azotemia, proteinuria and decreased urinary specific gravity (USG) [7, 8]; systolic blood pressure (SBP) may also rise as a consequence of renal disease [9].

The diagnosis of CanL is complex and should rely on information/history of a potential previous exposure to the parasite, clinical signs and laboratory findings, followed by confirmation of parasitic infection using parasitological, molecular and immunological techniques [3, 7, 10]. The LeishVet group developed a staging system which categorizes CanL in four stages, taking into account the clinical signs, clinicopathological findings and serological titres [8, 10]: mild (stage I), moderate (stage II), severe (stage III) and very severe (stage IV) disease. Stage II is divided in substages A (creatinine < 1.4 mg/dl and urinary protein-to-creatinine ratio [UPC] < 0.5) and B (creatinine < 1.4 mg/dl, but UPC = 0.5–1).

The most recommended antileishmanial protocols are the combination of allopurinol with meglumine antimoniate or miltefosine [10–13], and a recent study showed that most Portuguese veterinarians follow the recommended guidelines for antileishmanial treatment [14]. Nevertheless, little is known about the therapeutic approaches preferred by Portuguese veterinarians to manage concomitant renal disease, which is highly prevalent and the main cause of death/euthanasia in these canine patients, particularly in those dogs in latter stages of CanL.

Kidney disease in CanL should be classified and treated following the International Renal Interest Society (IRIS) recommendations [15, 16]. Roura et al. [9] recently recommended starting treatment with antileishmanial drugs only at UPC < 3.0 and to re-evaluate 4 weeks later. If proteinuria remains > 0.5 despite antileishmanial treatment, a renal diet should be started and eventually combined with an angiotensin-converting enzyme inhibitor (ACEI). Depending on the subsequent re-evaluations of proteinuria, the dose of ACEI may be increased and angiotensin receptor blockers (ARB) and polyunsaturated fatty acids can be added to the therapeutic regimen. Aldosterone receptor blockers are recommended in dogs with increased serum aldosterone concentrations which have not responded to or not tolerated ACEI and/or ARB [16]. Calcium channel blockers are also recommended as part of an antihypertensive treatment [16, 17].

The choice of standard antiproteinuric treatment in dogs remains a topic of discussion, but the use of immunosuppressants in dogs with immune-mediated glomerular disease is controversial [18]. However, the latter therapy is being increasingly accepted, taking into account that the patient is more likely to die from the consequences of glomerular disease than from the actual impact of the underlying infectious cause [17]. Mycophenolate mofetil is the recommended first-line drug to treat peracute or rapidly progressive renal disease, either alone or in combination with prednisolone [18], while mycophenolate mofetil, chlorambucil, azathioprine and cyclosporine are also considered for the treatment of stable or slowly progressive conditions [18]. Based on clinical experience, Roura et al. [9] suggested the use of an anti-inflammatory dosage of prednisone/prednisolone (0.7 mg/kg orally, once a day, over 3–10 days), justifying its use by the potential of these corticosteroids to reduce immune-mediated renal inflammation rather than decreasing the formation and circulation of immune complexes [9].

The aim of the present study was to assess how veterinary practitioners in Portugal currently treat proteinuria in dogs with CanL. We report the responses of participants in a survey, detailing drug prescription, dietary treatment and current trends on the use of immunosuppressants when there is suspected glomerular involvement.

## Methods

An online survey was developed and uploaded onto an electronic platform (Google Forms®). The survey included 46 multiple-choice and 18 open-ended questions on the diagnosis and medical management of CanL. Due to the number of questions and concurrent information extension, part of these results on diagnosis and medical management have already been published [14]. For the purpose of the present study, only those questions focusing on proteinuria and glomerular disease were considered. After validation by an epidemiologist, the survey was uploaded onto a mailing list of general veterinary practitioners for a 4-week period to determine how easy the questions were to answer and to identify practical mistakes. Thereafter, it was distributed on social media groups of veterinary practitioners working in Portugal for another 4 weeks. The questions analyzed in the present study are shown in detail in Additional file 1: Table S1.

Veterinarians who answered the survey were exposed in a virtual setting to three specific but theoretical scenarios of dogs living in areas endemic for CanL without any preventive measures (repellents/insecticides, vaccination or domperidone) that had various clinical and laboratory findings suggestive of CanL. All the cases had proteinuria. Specifically, the UPC in the three scenarios was 0.5, 1.2 and 3.5, respectively, allowing the respective cases, given the whole context, to be classified as LeishVet IIb, III and IV stages. Table 1 provides the details on each clinical scenario.

**Table 1:** Summary of the descriptions given in the questionnaire on each clinical case

Description	LeishVet classification		
	Stage IIb	Stage III	Stage IV
Signalment	Male, 6 years old	Male, 7 years old	Male, 12 years old
Iatrotropic stimulus	Epistaxis	Lethargy, anorexia, weight loss, PU/PD, auricular lesions	Lethargy, anorexia, weight loss, skin wounds, PU/PD
Physical examination	Epistaxis	Pale mucosae, generalised lymphadenomegaly, mucocutaneous ulcerative lesions, ears’ crusts	Pale mucosae, facial and plantar exfoliative dermatitis, onychogryphosis, nasal hyperkeratosis and ulceration
CBC, biochemical profile, urinalysis	Mild non-regenerative anaemia	Moderate non-regenerative anemia, hyperproteinemia, hypoalbuminemia	Moderate non-regenerative anemia, hyperproteinemia, hypoalbuminemia
Serum protein electrophoresis	Hyperglobulinaemia without hypoalbuminemia	Hyperglobulinemia with polyclonal gammopathy	Hyperglobulinemia with polyclonal gammopathy
Creatinine (mg/dl)	< 1.4	1.9	3.5
Urinalysis	UPC = 0.5; inactive sediment	USG = 1018; UPC = 1.2; inactive sediment	UPC = 6.2; inactive sediment
Serological titre	1:640	1:320	1:640
Other exams	-	Blepharitis, uveitis; US: splenomegaly; SBP: normal	Corneal opacification

*CBC* complete blood count, *PU/PD* polyuria/polydipsia, *SBP* systolic blood pressure, *UPC* urinary protein-to-creatinine ratio, *US* ultrasound

For each clinical case, veterinary practitioners were asked if they would treat the proteinuria and whether they would consider switching to a renal diet. In addition, they were asked to list in detail which compounds they used to manage proteinuria in daily practice. They were also asked if they combined drugs to treat proteinuria and, when the answer was affirmative, which combinations they used. To evaluate these items, we used a multiple-choice question that included options as: “ACEI,” “ARB,” “CCB,” “aldosterone receptor blockers,” “antithrombotic therapy” and “other(s)”. Only one option could be selected at first; but in a second question it was possible to choose one or more items. After the presentation of clinical scenarios, veterinarians were asked (yes or no) whether they commonly use immunosuppressants in dogs with suspected glomerular disease secondary to CanL.

All data were collected using Google Forms® and downloaded in a database (Microsoft Excel 2016®; Microsoft Corp., Redmond, WA, USA) for descriptive statistical analysis.

In the present article, the terms “respondent,” “clinician,” “practitioner” and “veterinarian” are used interchangeably (i.e. as synonyms).

## Results

A total of 86 veterinary practitioners responded to the survey and included in the analysis. Since no major problem areas were detected during the internal validation process, the questionnaire was the same during all 8 weeks of the survey, and answers collected during the first phase were included in the global descriptive statistical analysis.

### Antiproteinuric treatment

Facing a theoretical scenario of a dog with leishmaniosis at stages IIb, III and IV, 16.3% (14/86), 62.8% (54/86) and 93.8% (71/81) of the respondents, respectively, assumed they would consider antiproteinuric treatment. For the stage IV scenario, 5.8% (5/86) of the veterinary practitioners elected for euthanasia and, consequently, were not included in the analysis, with the result that this sample size for this scenario was 81 (Table 2).

**Table 2:** Willingness to treat proteinuria and to apply a renal diet in CanL stages IIb, III and IV

Questions	LeishVet classification		
	Stage IIb	Stage III	Stage IV
“*Would you treat proteinuria?*”	*n* = 86	*n* = 86	*n* = 81
No	72 (83.7%)	32 (37.2%)	5 (6.2%)
Yes	14 (16.3%)	54 (62.8%)	76 (93.8%)
“*Would you consider a renal diet?*”	*n* = 14	*n* = 54	*n* = 76
No	10 (71.4%)	9 (16.7%)	2 (2.6%)
Yes	4 (28.6%)	45 (83.3%)	74 (97.4%)

Among those respondents who assumed that they would manage proteinuria, 28.6% (10/14), 83.3% (45/54) and 97.4% (74/76) recommended switching to a renal diet with leishmaniosis stages IIb, III and IV, respectively (Table 2).

In addition to diet, clinicians detailed the preferred first-line antiproteinuric drug and the potential combination therapies performed in daily practice. The results are shown in Table 3.

**Table 3:** First-choice drugs and combined protocols chosen by respondents for the treatment of proteinuria in the clinical scenarios of CanL stages IIb, III and III

First-choice drugs and combined protocols	LeishVet classification		
	Stage IIb (*n* = 14)	Stage III (*n* = 54)	Stage IV (*n* = 76)
*a. First-choice drugs*^a^			
ACEI	14 (100%)	46 (85.2%)	60 (78.9%)
ARB	0%	5 (9.3%)	10 (13.2%)
CCB	0%	2 (3.7%)	4 (5.3%)
Antithrombotic therapy	0%	1 (1.9%)	2 (2.6%)
*b. Single-drug and combined protocols*^b^			
ACEI	9 (64.3%)	22 (40.7%)	35 (46.1%)
ACEI + NA	4 (28.7%)	7 (13.0%)	0%
ACEI + aldosterone receptor blockers	0%	1 (1.9%)	1 (1.3%)
ACEI + antithrombotic therapy	0%	6 (11.1%)	11 (14.5%)
ACEI + ARB	1 (7.1%)	4 (7.4%)	9 (11.8%)
ACEI + CCB	0%	2 (3.7%)	3 (3.9%)
ACEI + ARB + antithrombotic therapy	0%	0%	3 (3.9%)
ACEI + CCB + aldosterone receptor blockers	0%	0%	1 (1.3%)
ACEI + other	0%	5 (9.3%)	2 (2.6%)
* SUM (ACEI with other compounds)*	*1 (7.1%)*	*18 (33.3%)*	*30 (39.5%)*
ARB	0%	4 (7.4%)	6 (7.9%)
ARB + antithrombotic therapy	0%	1 (1.9%)	1 (1.3%)
CCB	0%	0%	4 (5.3%)
CCB + NA	0%	2 (3.7%)	0%
* SUM (protocols without ACEI)*	*0%*	*7 (12.9%)*	*11 (14.5%)*

*ACEI* angiotensin converting enzyme inhibitors angiotensin converting enzyme inhibitors, *ARB* angiotensin receptor blockers, *CCB* Calcium channel blockers, *NA* no answer/not detailed

^a^Only one option available

^b^One or more options available

In the theoretical scenario of the dog with leishmaniosis stage IIb, 100% (14/14) of the clinicians chose ACEI as their treatment of choice to treat proteinuria. Among these, 64.3% (9/14) opted to use it as a single-therapy, 28.6% (4/14) did not provide any details and 7.14% (1/14) replied that they would combine it with an ARB (Table 3).

Among the 54 respondents who would proceed with the medical management of proteinuria in the leishmaniosis stage III scenario, 85.2% (46/54) prioritized ACEI as a first-choice drug, followed by ARB (9.3%; 5/54), CCB (3.7%; 2/54) and antithrombotic therapy (1.9%; 1/54). Regarding potential therapeutic combinations, 40.7% (22/54) kept ACEI as monotherapy, and 13.0% (7/54), although electing for ACEI, did not provide any information on whether they would combine it with other drugs. A total of 33.3% (18/54) reported combining ACEI with other compounds, such as ARB, CCB, antithrombotic drugs, among others (Table 3). Associations which did not include ACEI were chosen by a total of 12.9% (7/54) of respondents.

Considering the theoretical scenario of the dog with leishmaniosis stage IV, only 81 answers were analysed, as five respondents were excluded from the analysis due to their preference for euthanasia. Among the 76 clinicians who reported treating proteinuria, 78.9% (60/76) elected for ACEI as first-choice treatment, followed by 13.2% (10/76) who preferred ARB. CCB and antithrombotic therapy were prioritized by 5.3% (4/76) and 2.6% (2/76) of the respondents, respectively. Regarding eventual drug combinations, ACEI was kept as monotherapy by 46.1% (35/76), while 14.5% (11/76) reported that they would combine it with antithrombotic drugs. Protocols using combinations of ACEI with other compounds were mentioned by 39.5% (30/76) of respondents. Therapies which did not include ACEI were reported by 14.5% (11/76) of respondents.

### Immunosuppressants in glomerular disease secondary to CanL

The use of immunosuppressants to treat glomerular disease secondary to CanL was considered by 44.2% (38/86) of the respondents; of these, prednisolone was chosen by 94.7% (36/38), while the remaining 5.3% (2/38) preferred mycophenolate mofetil. No other immunosuppressants were selected by the respondents.

Concerning the dosage of immunosuppressants, the two respondents answering that they would use mycophenolate mofetil mentioned that would use the “recommended dosage” and did not specify it. Among the 36 respondents reporting the use of prednisolone, 66.7% (24/36) would use the “recommended dosage” but did not provide any details. Regarding dosage, 0.5–1 mg/kg twice daily, 1 mg/kg twice daily and 1–2 mg/kg once daily would be administered by 2.7% (1/36) of the respondents, each. Dosages of 0.5, 1 and 2 mg/kg were mentioned by 2.6% (1/38), 21.1% (8/38) and 2.6% (1/38) of respondents, respectively, but no details on the frequency of administration were reported.

## Discussion

Using information provided by 86 veterinary practitioners who responded to an online questionnaire, we evaluated how veterinarians from Portugal, a country where CanL is endemic, approached the treatment of proteinuria in dogs with leishmaniosis. Specifically, we aimed to clarify the preferred medical management protocol and whether immunosuppressants are considered in the case of glomerular disease. When treating a dog with leishmaniosis, it is important not only to control the infection, but also to treat any complications that develop during the course of the disease, with renal disorders being among the most frequent of complications in such patients [4]. This survey included three virtual scenarios, corresponding to CanL stages IIb, III and IV, in which renal impairment was described. By analyzing the responses of veterinary practitioners to this questionnaire, we were able to assess how these practitioners deal with proteinuria in clinical practice.

With respect to the use of antiproteinuric treatment in daily practice, the results from this survey show that antiproteinuric treatment increased with increasing magnitude of proteinuria (and azotemia). According to some authors [9, 19, 20], given that proteinuria decreases within 4 to 8 weeks following the initiation of antileishmanial treatment, the CanL stages IIb and III scenarios in our survey could be treated only with antileishmanial drugs, and antiproteinuric compounds should only be considered 4 weeks later if the UPC remained > 0.5. However, in our survey, 16.3% and 62.8% of the veterinarians who responded stated that they would apply antiproteinuric treatment in combination with antileishmanial drugs in CanL stages IIb and III, respectively, while almost all respondents (93.8%) stated that antiproteinuric treatment would be appropriate in the CanL stage IV with UPC > 3.0 (creatinine = 3.5 mg/dl and UPC = 6.2 in our scenario). These results reflect that even in early stages of CanL, proteinuria is immediately addressed independently of antileishmanial protocols. As this study was conducted before the publication of a recent consensus on CanL and chronic kidney disease (CKD) [9], these findings reinforce the inconsistent approach on proteinuria management in daily veterinary practice. Although the publication of recent guidelines can in part contribute to a more homogeneous and step-by-step approach, particularly regarding the treatment of CanL stages IIb and III, the question of whether or not proteinuria should be addressed at the time antileishmanial therapy is initiated or only after 4 weeks of therapy remains controversial.

Regarding the antiproteinuric drugs chosen, ACEI were the preferred compounds. Indeed, ACEI are the most recommended compounds to treat proteinuria in dogs, along with a change in diet to a renal diet [9, 16–18, 21–23]. To a much lesser extent, some veterinarians selected ARB, CCB and antithrombotic drugs as first-line protocols, with the use of these compounds increasing in line with the severity of renal disease (and CanL). With the advent of ARB for the treatment of canine nephrology disorders [24–26], further studies are needed to clarify which drug is more appropriate for cases of CanL with glomerular involvement. The prescription of antithrombotic therapy in CanL stages III and IV may be justified by the hypoalbuminemia described in those scenarios, although guidelines [9, 16] recommend the use of this therapy when hypoalbuminemia is severe, which was not described in the hypothetical clinical cases in this survey.

In addition to the use of pharmacological treatment for CanL, the choice for a renal diet was also seen to have become increasingly accepted therapy, in line with the worsening of renal disease. Recommendations in the most recent literature [9, 16] are that CanL stage IIb would only require monitoring in addition to antileishmanial treatment. According to Roura et al. [9], CanL stage III should first be monitored, given that UPC is < 3.0, and antiproteinuric treatment should only be considered at follow-up, 4 weeks later. Nevertheless, such protocols may vary according with the clinical status of the patient and should be applied on a per-patient basis [9]. Therefore, it is possible that diet is unnecessarily changed in the treatment of earlier CanL stages, in which, following recent guidelines, proteinuria should be assessed 4 weeks after the onset of antileishmanial treatment.

Even though our results are generally in accordance with the recommendations, the use of non-recommended protocols showed some inconsistency and misinformation among the respondents regarding the management of proteinuria in dogs with leishmaniosis, especially in those with lower UPC values. These results stress the need to increase awareness of the role of medical management in proteinuria.

The use of immunosuppressants in dogs suspected of glomerular disease secondary to leishmaniosis is particularly controversial, as demonstrated by the respondents falling approximately evenly (50%) into the two categories (yes/no), emphasizing the lack of agreement in such cases. The controversy usually focuses on the possibility that these drugs may compromise the immune response against infection and worsen the clinical status rather than help reducing the immune-mediated inflammation. Among those respondents who reported using immunosuppressants, almost all prioritized prednisolone and a small proportion chose mycophenolate mofetil. These results are in contrast with the consensus recommendations for the treatment of immune-mediated glomerular disease [18], given that glucocorticoids have considerable adverse effects (such as worsening of proteinuria and hypertension); as such, mycophenolate mofetil is the recommended first-choice immunosuppressant for these cases. Nonetheless, the latest recommendations state the use of prednisone at an anti-inflammatory dosage as an effective approach [9]. Only 7.9% (3/38) of the respondents provided details on the dose and frequency of administration used; therefore, it was not possible to properly evaluate this information. Although a recent study [9] reported the use of prednisolone at 0.7 mg/kg once daily in cases of suspected glomerular disease, further studies are required to clarify the best recommended dose for its use in these patients. These results and the incongruent position of immunosuppressants in the medical management of CanL stress the need for comparative studies to clarify whether immunosupressants are recommended and, if so, which compound provides the best effect.

This study had several limitations that need to be considered. The number of replies (*n* = 86) was relatively small, considering the estimated number of veterinarians registered in Portugal [27]. However, the number of those actively working as small-animal practitioners and belongs to the network groups where the questionnaire was distributed is unknown, and the number of replies is in line with those reported in other studies [28, 29]. Another limitation was that some details on the clinical cases were absent, with the aim to limit the size of the questionnaire.

Although a recent publication on guidelines addressing the main problems of glomerular disease in dogs with leishmaniosis is helpful [9], this survey provides a better understanding on how veterinarians currently manage proteinuria in clinical practice.

## Conclusions

This study collects and provides useful information on the management of renal disease, one of the most common and important complications of CanL in daily practice. According to the responses, in terms of antiproteinuric treatment, pharmacological and dietary therapeutic protocols are increasingly prescribed in line with the worsening of proteinuria. The choice for ACEI by the large majority of responders as a first-choice drug and the administration of a renal diet, especially in CanL stage IV, showed that these Portuguese veterinary practitioners were aware of the current, most adequate protocols and international recommendations given by expert groups in leishmaniosis and renal disease. Finally, the present study stresses the discrepancies and lack of consensus and scientific evidence supporting the use of immunosuppressants when glomerular disease secondary to CanL is suspected. In addition to clarifying how veterinarians currently manage proteinuria on a daily basis, this study provides new insights into current incongruencies on the use of immunosuppressants, stressing the need of further studies to better prove the benefits or disadvantages of this therapy.

## Supplementary Information

**Additional file 1: Table S1**. Questionnaire provided online to veterinarians: “Management of canine leishmaniosis in Portugal: questionnaire-based survey”.

## Data Availability

The datasets supporting the conclusions of this article are included within the article (and in Additional file 1: Table S1).

## Abbreviations

ACEI: Angiotensin-converting enzyme inhibitors
ARB: Angiotensin receptor blockers
CanL: Canine leishmaniosis
CBC: Complete blood count
CKD: Chronic kidney disease
PU/PD: Polyuria/polydipsia
SBP: Systolic blood pressure
UPC: Urinary protein-to-creatinine ratio
US: Ultrasound
USG: Urinary specific gravity
