# Supplementary material for: Dogs with leishmaniosis: how are we managing proteinuria in daily practice? A Portuguese questionnaire-based study
Source: Parasit Vectors. 2022 Apr 11;15:125. doi: 10.1186/s13071-022-05222-w (PMC8996528; doi:10.1186/s13071-022-05222-w)
Supplement: Supplementary file 1 — Additional file 1: Table S1. Questionnaire provided online to veterinarians: “Management of canine leishmaniosis in Portugal: questionnaire-based survey”. [file 13071_2022_5222_MOESM1_ESM.docx]

**Questionnaire provided online – English version**

**Management of canine leishmaniosis (CanL) in Portugal – diagnosis and treatment**

To whom it may concern,

If you are a veterinarian working in Portugal, in small animal clinic – namely, with dogs – then, this questionnaire is for you!

This questionnaire consists, essentially, of three parts: “Veterinarian profile”, “Clinical cases” and “Other questions”, always related to CanL.

The questionnaire contains around 50 questions and will take approximately 15 minutes to complete. It is worth noting that the answers are anonymous and will be analysed with proper confidentially, thus we kindly ask you to answer honestly, to allow obtaining valid and representative conclusions. Data privacy will be equally preserved in case of those being used in written or oral communications, or in publications that may result from the study.

In case of any questions related to this questionnaire, please contact us. More information concerning the study will be sent to the participants, via email, if it is their willing. In that case, please send an email showing your interest.

Thank you for your collaboration and time spent replying to this questionnaire!

**FIRST PART – VETERINARIAN PROFILE**

(…)

**SECOND PART – CLINICAL CASES**

Three clinical cases will be presented, always related to CanL. Answers should be given bearing in mind your clinical practice context and knowledge on this topic.

**1^st^ Clinical case**

Dog, male, 7 years old, living in a geographic area where CanL is endemic and does not receive any preventive measures for the infection/disease (insecticides, vaccine, immunomodulator).

**Reason for consultation**: prostration, anorexia, weight loss, polyuria/polydipsia, auricular lesions.

**General examination:** Pale mucosae, generalised lymphadenomegaly, mucocutaneous ulcerative lesions, ears’ crusts.

**Ophtalmologic exam:** blepharitis, uveitis (bilateral intraocular pressure reduction, erythema).

**Abdominal ultrasound:** splenomegaly.

**Laboratory tests:** Moderate nonregenerative anaemia, hyperproteinemia, hypoalbuminemia, hyperglobulinemia with polyclonal gammopathy.

Mild azotaemia (creatinine 1.9 mg/dL); urinalysis: USG = 1.018, proteinuria (UPC = 1.2) with inactive sediment.

Normal systemic blood pressure.

**Serology (immunofluorescence):**

Anti-*Leishmania* antibody titre: positive for 1:320 dilution.

Negative serology for other concomitant CVBD agents (*Anaplasma* spp.*, Babesia* spp.*, Ehrlichia* spp.*, Borrelia* spp.*, Rickettsia* spp.*, Hepatozoon* spp.*, Hemobartonella canis, Bartonella* spp.*, Dirofilaria* spp.*, Trypanosoma* spp.*)*.

**Reference serological values:**

Cut-off: positive anti-*Leishmania* antibody titres at 1:80 dilution.

1. Considering that this animal had a UPC of 1.2 with inactive sediment, would you treat proteinuria in this case?

□ No □ Yes

1. If yes, which treatment would you prefer to apply?

□ Calcium channel blockers (CCB) (e.g. amlodipine)

□ Aldosterone receptor blockers (e.g. spironolactone)

□ Angiotensin receptor blockers (ARB) (e.g. losartan, telmisartan)

□ Angiotensin-converting enzyme inhibitors (ACEI) (e.g. enalapril, benazepril)

□ Other

□ Antithrombotic therapy (e.g. aspirin, clopidogrel)

1. In case you selected “Other”, which drug is it?
2. Besides the one selected as preferential, would you add any other drug(s)? If yes, which one(s)?

□ Calcium channel blockers (CCB) (e.g. amlodipine)

□ Aldosterone receptor blockers (e.g. spironolactone)

□ Angiotensin receptor blockers (ARB) (e.g. losartan, telmisartan)

□ Angiotensin-converting enzyme inhibitors (ACEI) (e.g. enalapril, benazepril)

□ I would not add any other drug

□ Other

□ Antithrombotic therapy (e.g. aspirin, clopidogrel)

1. Would you start a renal diet?

□ No □ Yes

**2^nd^ Clinical case**

Dog, male, 6 years old, living in a geographic area where CanL is endemic and does not receive any preventive measures for the infection/disease (insecticides, vaccine, immunomodulator).

**Reason for consultation**: Epistaxis.

**General examination:** No abnormalities.

**Laboratory tests:** Mild nonregenerative anaemia. Hyperglobulinaemia without hypoalbuminemia. Creatinine <1.4 mg/dL (normal), borderline proteinuria (UPC = 0.5), inactive sediment.

**Serology (immunofluorescence):**

Anti-*Leishmania* antibody titre: positive for 1:640 dilution.

Negative serology for other concomitant CVBD agents (*Anaplasma* spp.*, Babesia* spp.*, Ehrlichia* spp.*, Borrelia* spp.*, Rickettsia* spp.*, Hepatozoon* spp.*, Hemobartonella canis, Bartonella* spp.*, Dirofilaria* spp.*, Trypanosoma* spp.*)*.

**Reference serological values:**

Cut-off: positive anti-*Leishmania* antibody titres at 1:80 dilution.

1. Considering that this animal had a UPC = 0.5 with inactive sediment, would you treat proteinuria in this case?

□ No □ Yes

1. If yes, which treatment would you prefer to apply?

□ Calcium channel blockers (CCB) (e.g. amlodipine)

□ Aldosterone receptor blockers (e.g. spironolactone)

□ Angiotensin receptor blockers (ARB) (e.g. losartan, telmisartan)

□ Angiotensin-converting enzyme inhibitors (ACEI) (e.g. enalapril, benazepril)

□ Other

□ Antithrombotic therapy (e.g. aspirin, clopidogrel)

1. In case you selected “Other”, which drug is it?
2. Besides the one selected as preferential, would you add any other drug(s)? If yes, which one(s)?

□ Calcium channel blockers (CCB) (e.g. amlodipine)

□ Aldosterone receptor blockers (e.g. spironolactone)

□ Angiotensin receptor blockers (ARB) (e.g. losartan, telmisartan)

□ Angiotensin-converting enzyme inhibitors (ACEI) (e.g. enalapril, benazepril)

□ I would not add any other drug

□ Other

□ Antithrombotic therapy (e.g. aspirin, clopidogrel)

1. Would you start a renal diet?

□ No □ Yes

**3^rd^ Clinical case**

Dog, male, 12 years old, living in a geographic area where CanL is endemic and does not receive any preventive measures for the infection/disease (insecticides, vaccine, immunomodulator).

**Reason for consultation**: lethargy, anorexia, weight loss, skin wounds, polyuria/polydipsia.

**General examination:** Pale mucosae, facial and plantar exfoliative dermatitis, corneal opacity, onychogryphosis, nasal hyperkeratosis and ulceration.

**Laboratory tests:** Moderate nonregenerative anaemia; hyperglobulinemia with polyclonal gammopathy, hypoalbuminemia. Azotaemia (creatinine 3.5 mg/dL), proteinuria (UPC = 6.2), inactive sediment.

**Serology (immunofluorescence – IFAT):**

Anti-*Leishmania* antibody titre: positive for 1:640 dilution.

Negative serology for other concomitant CVBD agents (*Anaplasma* spp.*, Babesia* spp.*, Ehrlichia* spp.*, Borrelia* spp.*, Rickettsia* spp.*, Hepatozoon* spp.*, Hemobartonella canis, Bartonella* spp.*, Dirofilaria* spp.*, Trypanosoma* spp.*)*.

**Reference serological values:**

Cut-off: positive anti-*Leishmania* antibody titres at 1:80 dilution.

1. Considering that this animal had a UPC = 6.2 with inactive sediment, would you treat proteinuria in this case?

□ No □ Yes

1. If yes, which treatment would you prefer to apply?

□ Calcium channel blockers (CCB) (e.g. amlodipine)

□ Aldosterone receptor blockers (e.g. spironolactone)

□ Angiotensin receptor blockers (ARB) (e.g. losartan, telmisartan)

□ Angiotensin-converting enzyme inhibitors (ACEI) (e.g. enalapril, benazepril)

□ Other

□ Antithrombotic therapy (e.g. aspirin, clopidogrel)

1. In case you selected “Other”, which drug is it?
2. Besides the one selected as preferential, would you add any other drug(s)? If yes, which one(s)?

□ Calcium channel blockers (CCB) (e.g. amlodipine)

□ Aldosterone receptor blockers (e.g. spironolactone)

□ Angiotensin receptor blockers (ARB) (e.g. losartan, telmisartan)

□ Angiotensin-converting enzyme inhibitors (ACEI) (e.g. enalapril, benazepril)

□ Other

□ Antithrombotic therapy (e.g. aspirin, clopidogrel)

1. Would you start a renal diet?

□ No □ Yes

**THIRD PART – OTHER QUESTIONS**

This third and last part refers to the current guidelines concerning diagnosis and treatment of CanL.

1. Do you use immunosuppressants when suspecting of glomerulonephritis secondary to CanL?

□ No □ Yes

1. If yes, which would be your first choice?

| □ Azatioprine  □ Cyclophosphamide | □ Cyclosporine  □ Chlorambucil | □ Micophenolate mofetil  □ Prednisolone | □ Other |
| --- | --- | --- | --- |

1. In case you selected “Other”, which treatment is it?
